# Implementation framework for AI deployment at scale in healthcare systems

**DOI:** 10.1016/j.isci.2025.112406

**Published:** 2025-04-11

**Authors:** Hassan Sami Adnan, Amitis Shidani, Lei Clifton, Clare R. Bankhead, Rafael Perera-Salazar

**Affiliations:** 1 Nuffield Department of Primary Care Health Sciences, University of Oxford, Oxford, UK; 2 Department of Statistics, University of Oxford, Oxford, UK; 3 Nuffield Department of Population Health, University of Oxford, Oxford, UK

**Keywords:** Public health, Artificial intelligence, Machine learning

## Abstract

Artificial intelligence (AI) and digital health technologies are increasingly used in the medical field. Despite promises of leading the future of personalized medicine and better clinical outcomes, implementation of AI faces barriers for deployment at scale. We introduce a novel implementation framework that can facilitate digital health designers, developers, patient groups, policymakers, and other stakeholders, to co-create and solve issues throughout the life cycle of designing, developing, deploying, monitoring, and maintaining algorithmic models. This framework targets health systems that integrate multiple machine learning (ML) models with various modalities. This design thinking approach promotes clinical utility beyond model prediction, combining privacy preservation with clinical parameters to establish a reward function for reinforcement learning, ranking competing models. This allows leveraging explainable AI (xAI) methods for clinical interpretability. Governance mechanisms and orchestration platforms can be integrated to monitor and manage models. The proposed framework guides users toward human-centered AI design and developing AI-enhanced health system solutions.

## Graphical abstract

**Figure:**
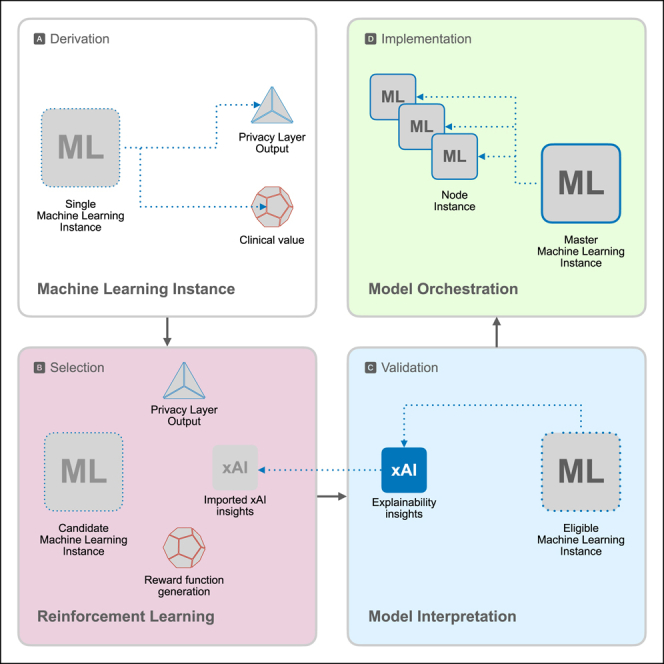

## Introduction

With the advancements in machine learning (ML), deep learning (DL), and the adoption of big data, medical artificial intelligence (AI) has become a rapidly growing field in medicine. This growth is fueled by the increased digitization efforts and the recognized potential of AI-enhanced solutions for better health and care outcomes.^1^^,^^2^^,^^3^ The ubiquitous concept of integrating algorithmic models based on AI in the development of healthcare is broadly categorized as digital health. Its necessity is well established, with policies and guidelines already being developed throughout the world.^4^^,^^5^^,^^6^^,^^7^^,^^8^^,^^9^ However, it still appears difficult to implement AI in clinical practice, medical research, and the broader healthcare fields, particularly when the focus is on clinical utility.^10^ Recent exponential growth of AI-related resources in the medical literature shows strong evidence of its beneficial utility for personalized medicine.^2^^,^^11^ Despite this growth, its translation into clinical settings with demonstrative evidence of clinical impact on patient outcomes remains unclear,^11^^,^^12^ while very few qualify as the clinical standard of practice.^13^ There are valid concerns, as discussed by experts in the field,^14^^,^^15^ about the promises of AI, with many of the expectations created still seeming unrealistic. Nonetheless, AI-enhanced tools have achieved human-level performance in areas like medical data analysis applications.^16^^,^^17^ The promise is that by automating routine clinical tasks and utilizing AI tools, physicians will spend valuable time with their patients, strengthening the inter-human bond that is based on trust, clinical presence, empathy, and communication.^3^^,^^17^

It is yet to be demonstrated that AI deployment has performed adequately and with reproducible accuracy in a broader healthcare setting.^12^ Effective healthcare practice goes beyond solving technical difficulties and requires an intimate understanding of the physician-patient social contract, geography-specific barriers to care (whether that be technological, linguistic, or policy related), and extraneous factors such as hospital-specific policies; these are factors which often cannot be accurately predicted.^10^^,^^18^ Among the many reasons why implementing AI-enhanced solutions faces difficulties is the gap from model accuracy to clinical efficacy – a manifestation described by Keane & Topol^19^ as the “AI chasm”. Currently, models are evaluated against several ML performance indicators, as no single measure exists to define model performance. However, such measures are restricted to the model itself and do not consider its clinical utility – the purpose, right use cases, and patient needs.^10^ Another barrier to implementation is trust, a key component to the patient-physician interaction, underpinning inter-human bonds that also apply to digital health tools and technologies.^11^ Furthermore, trust is an integral part of systems and services, with increasingly more decisions made based on trust,^11^^,^^16^^,^^20^ including when engaging communities and stakeholders to co-create solutions in healthcare.^21^ Safe and trustworthy AI requires accountability, data privacy, ethics, explainability, governance, and transparency.^4^^,^^22^^,^^23^^,^^24^^,^^25^^,^^26^ The list of barriers is even more extensive when we consider the broader healthcare systems and their interactions with other stakeholders. Related barriers include capacity building for AI implementation and transformation of healthcare roles and practices.^18^ The extent encompasses barriers that may not be immediately apparent; such examples include integration into the clinical workflow, data availability and its quality, finance and resources, and policy and regulation.^27^ Adding to these complex challenges related to the healthcare domain, the development process of technology itself is a complex task. This is why proponents of good software design always recommend prioritizing a well-designed approach as a first step.^28^^,^^29^^,^^30^^,^^31^ Systems, including healthcare, rely on dynamic change, and modularity becomes an integral part and an important strength,^32^ allowing the system to adapt to necessary change as system requirements constantly update. Framing the problem and having a guideline helps develop solutions that are part of a large network, making it manageable and easier to isolate problems of the modular part of the system without interrupting the other parts.^32^^,^^33^

Medical AI promises a future of personalized medicine and better clinical outcomes. However, the clinical requirements for the implementation of AI make this challenging; tailored solutions and designs are needed to meet these requirements. This paper presents the *Health xAI Implementation Framework*, which is intended for digital health stakeholders. It provides technical guidance for designing a full system based on AI-enhanced digital health tools and solutions. Following this framework, digital health designers will be able to tailor solutions based on the needs of the end users, while allowing developers to implement methods that coincide with healthcare requirements. Furthermore, it allows multisectoral co-creation and empowerment^21^ by providing provisions to integrate collaborative decisions in the entire design, development, deployment, monitoring, and management phases.

On a broader scope, the framework:

(1) presents a coherent plan for the integration of AI models and tools into the healthcare system,
(2) makes explicit the inclusion of other parameters (costs, interpretability, privacy, etc.) and links them with clinical outputs for the selection of AI models and tools,
(3) and provides a strategy for the evaluation in real-time of AI tools as well as for the approach to update or replace them within the system.

### The Health xAI Implementation Framework

**Figure 1:**
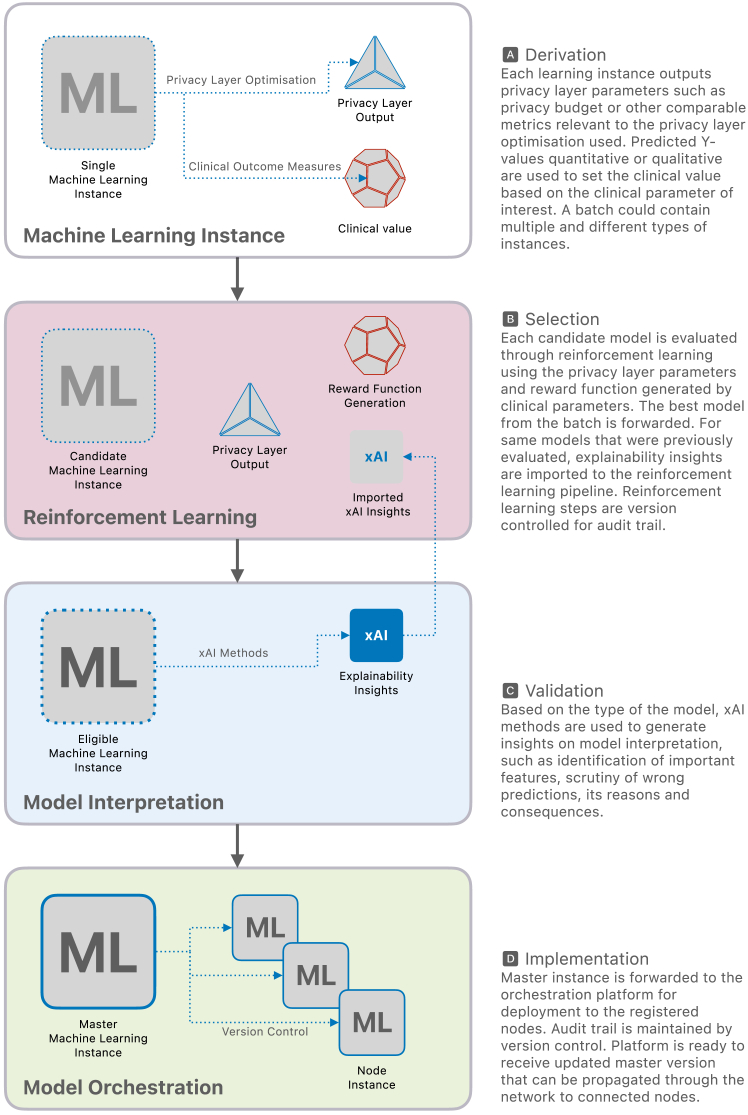
The Health xAI Framework

The *Health xAI Implementation Framework* (Figure 1) is an outline of a complex and adaptable decision-making implementation framework using bandit learning (BL)^34^ for optimizing combinations of AI/ML models based on multiple important metrics. Bandit learning is a method that uses reinforcement learning to carefully balance exploration – trying out new options – with exploitation – relying on the best-known choices – to find the best option in a given set. This approach aligns with the broader field of multi-objective optimization^35^ and decision-making in machine learning, where the aim is to find the best trade-offs between multiple conflicting objectives. In the proposed framework, BL is used to evaluate and select models based on a privacy layer output and clinical value (see *framework components*). This approach is particularly suitable for the static decision environment of the framework, where the goal is to select the best model from a fixed set of competing candidates. Compared to other RL algorithms, such as Q-learning or policy gradient methods, BL offers significant advantages in terms of computational simplicity, scalability, and efficiency in optimizing immediate rewards.^34^ While other RL methods are well-suited for dynamic environments with sequential decision-making, BL aligns better with the framework’s focus on iterative, multi-objective optimization. Its ability to handle multiple competing metrics with minimal computational demands ensures timely and actionable insights, which can be crucial in healthcare settings. The framework consists of 4 main components that work together to provide digital health designers with technical guidance for implementing predictive models at scale.^36^ The framework integrates: (1) a privacy-preserving infrastructure while enabling clinical outcome measures to be considered; (2) utilizes reinforcement learning techniques, such as bandit learning, to select the best models; (3) creates provisions for model interpretation; and (4) lays out the dynamics of design, development, deployment, monitoring, and maintenance of the models across multiple sites for scalability.

**Figure 2:**
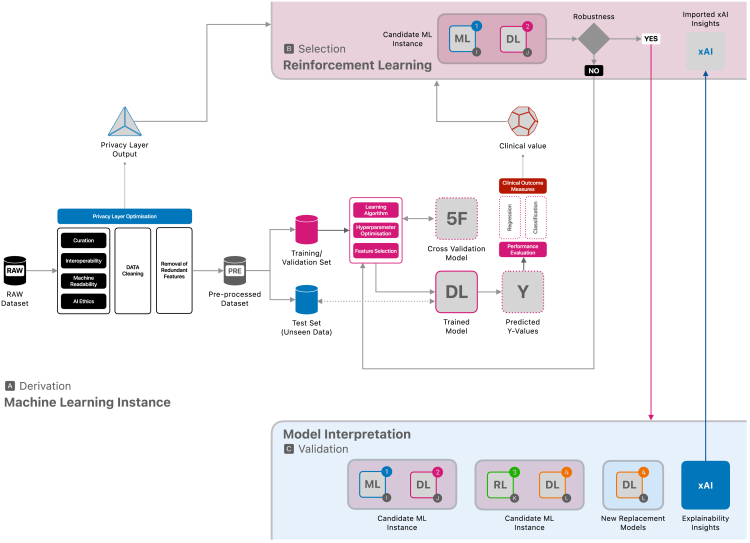
A single machine learning instance and its interaction within the Framework

**Figure 3:**
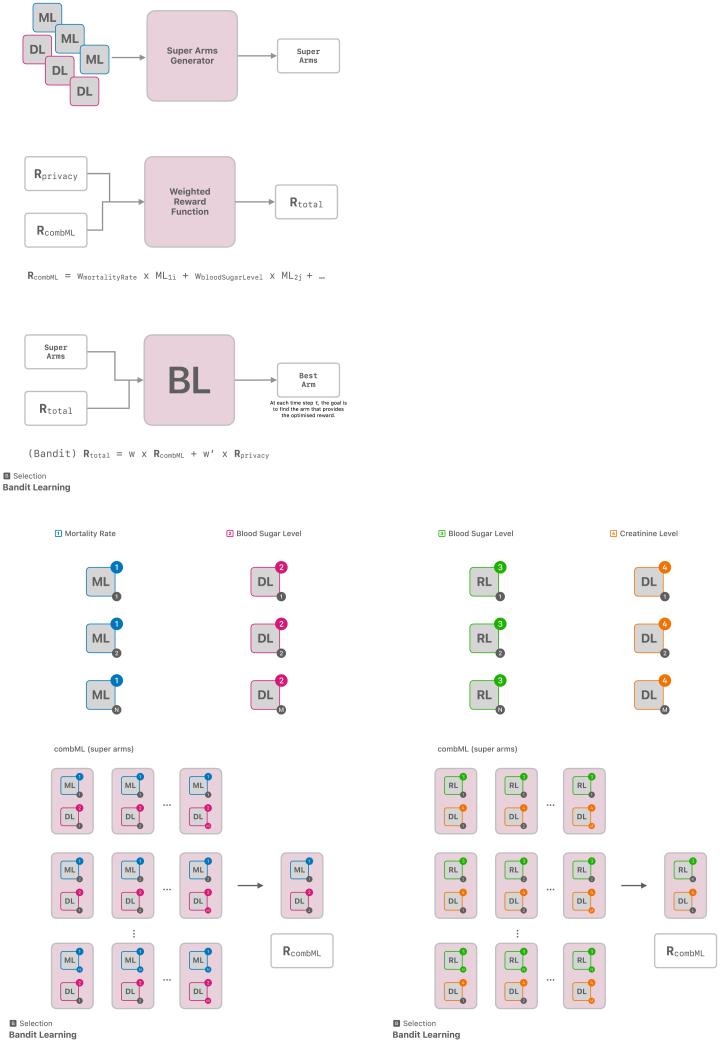
Creating the best arm based on each super arm and **R**_total_ that comprises **R**_privacy_ and **R**_combML_. Creating n-tuple super arms to define reward function **R**_combML_ in two different sites

**Figure 4:**
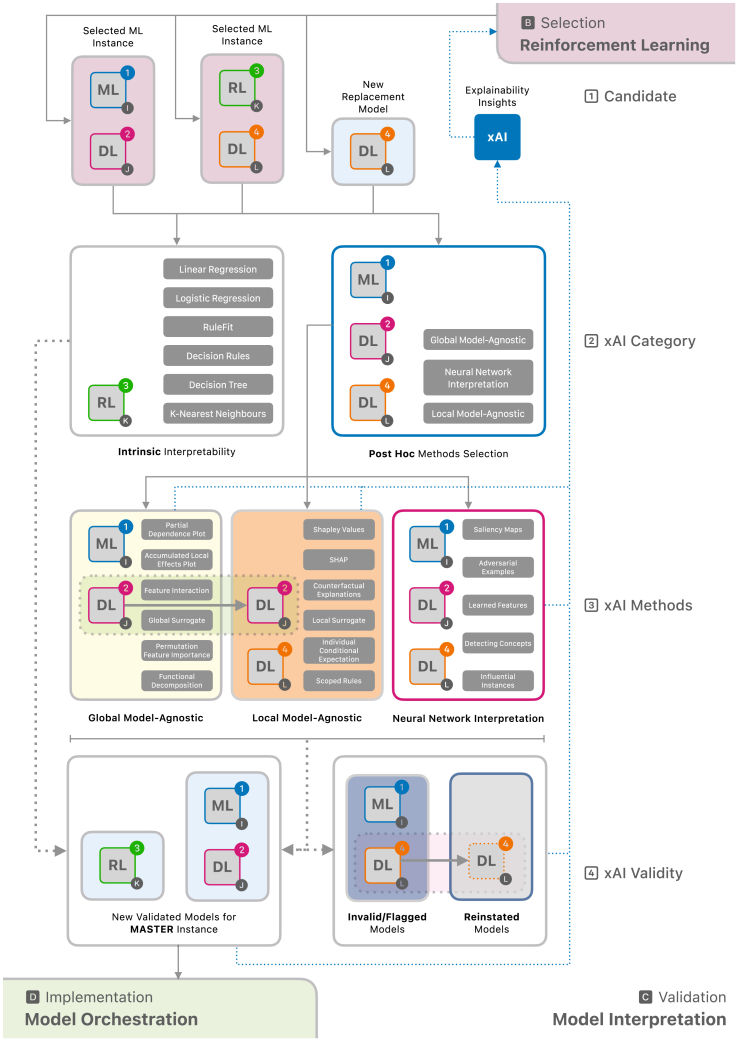
Comprehensive pipeline for model interpretation for xAI in healthcare

**Figure 5:**
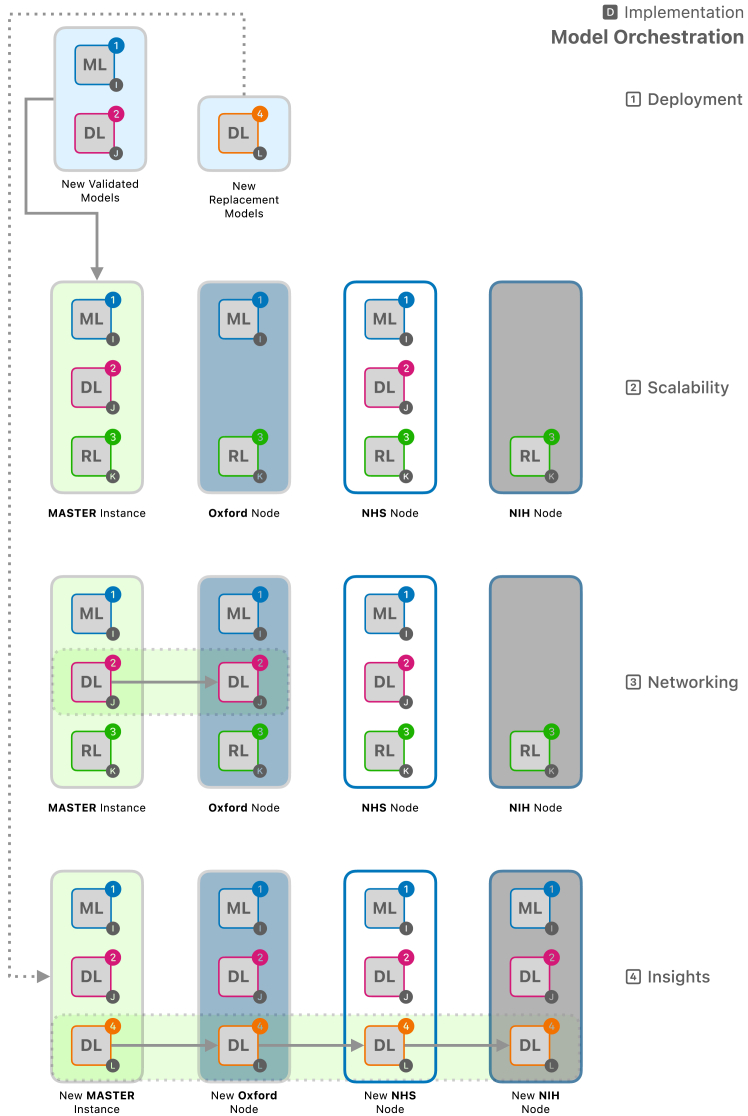
Orchestration platform to manage the implementation of xAI models at scale

Data privacy and data protection are priorities in deploying AI-enhanced healthcare solutions. A privacy-preserving infrastructure safeguards sensitive patient data while AI models analyze it or train on it. It employs techniques to achieve a balance between the need for precise model predictions and the ethical and legal obligations to protect patient confidentiality. There are already algorithms such as differential privacy^7^ that have been implemented in developing AI/ML methods,^37^^,^^38^ which are suitable for application in healthcare settings. The algorithm helps ensure that any analyses of the data would result in the same outcome even if a particular patient’s information were removed. This works as a guarantee that the contribution of any single patient remains untraceable. This integration of a privacy-preserving infrastructure means that privacy considerations are part of the design, development, deployment, monitoring, and maintenance life cycle and technical infrastructure of algorithmic models. Privacy-preserving data sharing has also been a focus for primary care researchers where a workaround has been practiced based on reciprocity, principles, and best practices,^20^^,^^39^ but would benefit from the integration of a technical solution that considers privacy as a built-in feature. It is, therefore, that this framework, for the first time to our knowledge, integrates a *‘Privacy Layer Optimization’* step in building algorithmic models and leveraging its output to be used as a reward function for bandit learning (Figures 1, 2, 3, 4, and 5), and provides provisions for integration of xAI methods. The *Privacy Layer Optimization* ensures that an AI/ML model can access a minimal amount of identifiable patient information. For example, if a model needs to classify patients into risk categories, the privacy layer will ensure that this classification is done without directly exposing sensitive details, such as the exact dates of medical events or specific laboratory results. Another example can be that when training a model to predict hospital readmission rates, the privacy layer optimization would introduce small random variations to patient data, ensuring that analyses reflect overall trends without exposing anyone’s exact information. For any model, the performance depends on but is not limited to, the pre-processing steps. Understanding the data structure and feature engineering while ensuring interoperability and machine readability is crucial for the output and the quality of explainability.^2^^,^^12^^,^^40^ To ensure the best privacy preservation and derive the necessary privacy layer output for the next components of the framework, the privacy optimization should be integrated throughout the entire pre-processing phase (Figure 2). For healthcare professionals, authorities and management, and patients, this means that patient data remains confidential even as AI models can access data that can lead to building tools for diagnosing conditions or predicting treatment outcomes. The privacy measures are built into the system from the start, reducing the risk of data breaches or misuse. This customizable (and updatable) nature of the setup allows flexibility to tailor solutions based on the variable and contextual requirements for healthcare systems. By embedding these principles into the AI workflow, we ensure that healthcare professionals can confidently use AI tools without compromising patient trust or compliance with data protection laws.

**Figure 6:**
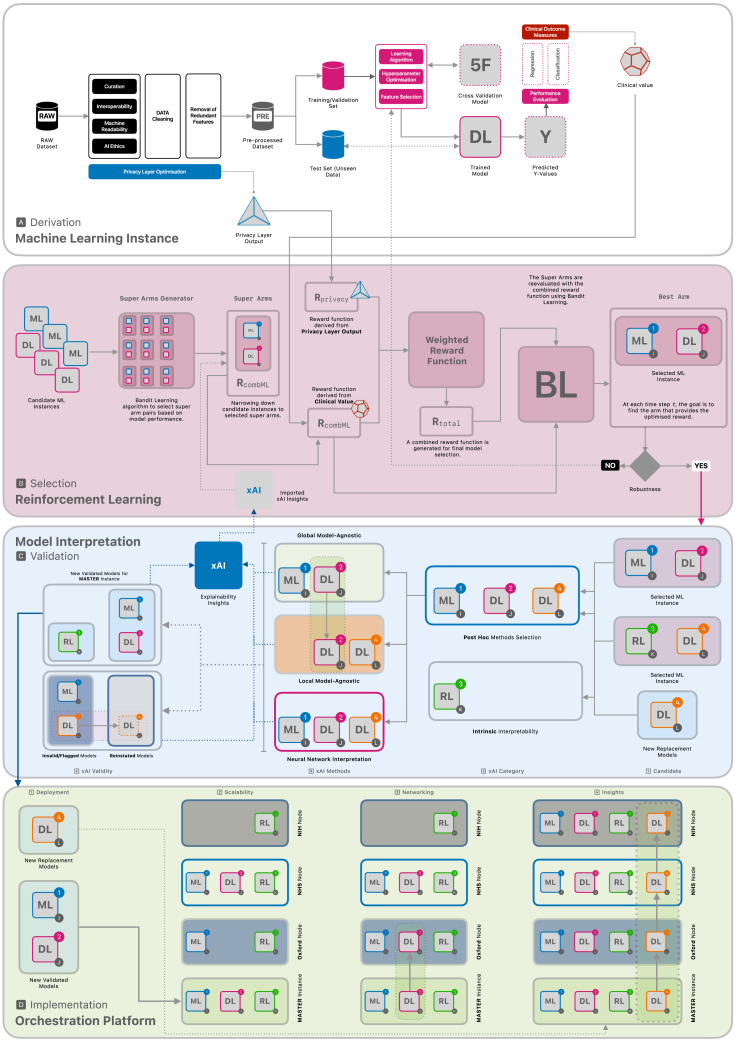
Orchestration platform to manage the implementation of xAI models at scale

**Figure 7:**
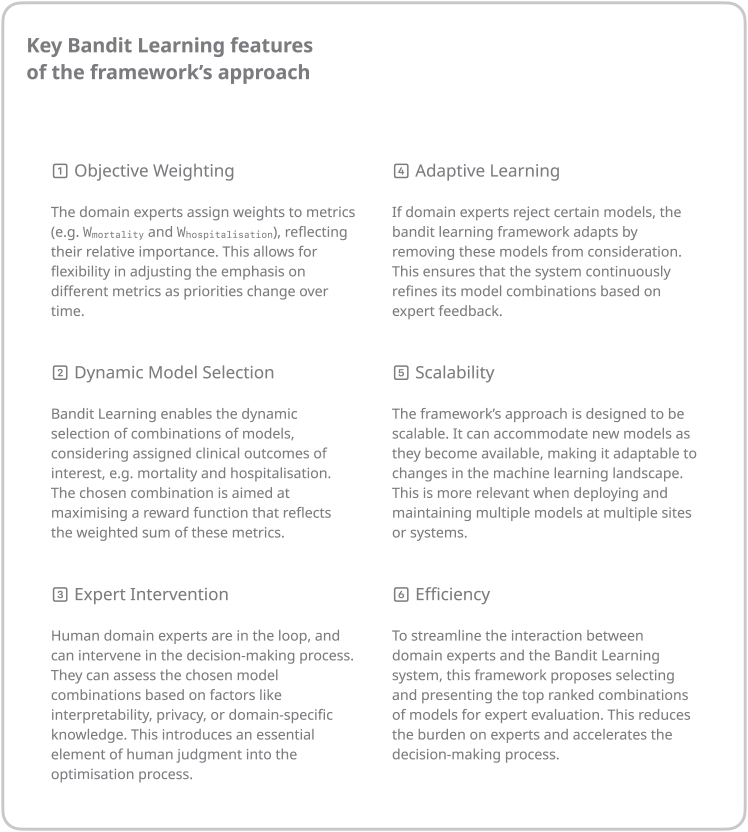
Key bandit learning features of the framework’s approach

**Figure 8:**
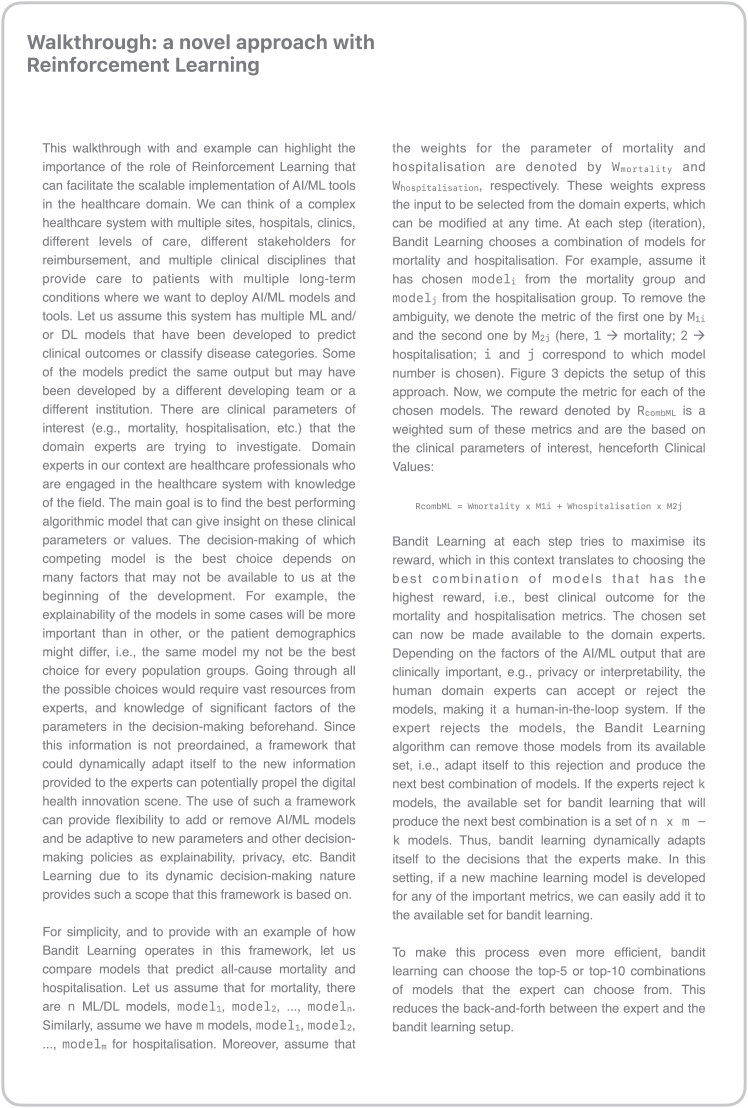
Walkthrough: A novel approach with reinforcement learning

Furthermore, early integration and built-in considerations of utilizing explainable AI (xAI) methods can strengthen AI governance strategies for designers and developers of algorithmic models based on different ML approaches. AI/ML tools can have a more significant clinical impact if clinicians and multiple stakeholders could interpret the output based on the type of explanation they need.^41^ Figure 6 illustrates the framework with a detailed pipeline and workflow as an example. Please refer to Figure 7 to see which bandit learning features are particularly important for this framework, and Figure 8 for a walkthrough with an example of how a novel reinforcement learning approach is utilized for this framework.

### Framework components

#### Derivation

A predictive model can be based on many methods, e.g., machine learning, deep learning, or reinforcement learning models, which generate two outputs (Figure 2), a *Privacy Layer Output* and a *Clinical Value*. These outputs are then used in the ‘Selection’ component of the framework. The privacy layer output is generated based on the privacy layer optimization relevant to the model and clinical/system context. This output value can be the privacy budget or any comparable loss function, depending on the use case and the privacy-preservation method used.^38^^,^^42^ The privacy budget acts as a limit, ensuring the system carefully manages how much data can be used for analysis while maintaining confidentiality. The use of the privacy budget can be tracked with version control to address and manage security concerns. The clinical value is generated from the predicted values of the models (quantitative or qualitative), which could be a constraint or pre-defined clinical outcome measures based on the clinical parameter of interest. During this pre-processing step, being aware of issues regarding data curation, interoperability, machine readability, and AI ethics will benefit the overall prerequisites for implementing models in the development life cycle.

The derivation component can contain a single or multiple models with different predictive model instances. All these models are candidates for the selection component.

#### Selection

The model selection is done utilizing bandit learning methods^34^ in three steps (Figure 3). Step one takes every candidate model initially given in the derivation component and creates best arms^43^ generating R_combML_ from super arms combML pairs. These combML pairs are every possible combination of available models that are being selected. In the context of Figure 3, the superscripts denote the different types of models, while the subscripts represent the various versions or instances of each model type. Step two combines the outputs from the derivation component to generate a reward function (R_total_) necessary for bandit learning.^34^ The privacy layer output defines R_privacy_, and the clinical values defines R_combML_. The R_total_, the combined reward function, is used with the super arms to select the best arm in step three. The decision-making of the selection mechanism through bandit learning enables the framework to keep the entire history (H_t_), and version control can provide an audit trail. This preserves information on the previously selected (best) arms, the total reward function associated with the best arms, and all the possible arms at that iteration. The output of the bandit learning model could also be a ranked list of arms.^43^ Here, ranking is based on the optimization problem taken from the reward function.

#### Validation

Once the best arms (eligible models) are proposed in the selection component, they are ready for model interpretation (Figure 4). Here, different xAI methods can be used depending on the model type and the scope of relevant interpretability. In Figure 4, the superscripts denote the different types of models, while the subscript I refers to the I-th model within the pool of machine learning candidates. Similarly, subscripts J, K, and L follow the same convention as their respective contexts. Models that do not fulfill the interpretability requirements are flagged and demoted in the Selection component so that they are no longer considered candidate models in the next bandit learning iteration. If *k* number of non-explainable super arms are found, the input for bandit learning for the next iteration would be *n* × *m* − *k* arms. Each time step *t* is logged in the history (H), and therefore, can be tracked back, and the removal of the ineligible candidates does not affect the bandit learning. This approach of disqualifying non-explainable models avoids the complication that could arise if Delayed Bandits^34^ were used where the R_total_ calculation with R_combML_ and R_privacy_ would require another additional factor of R_xAI_ as a delayed part to account for the ineligibility of the model based on the interpretability validation. This approach, however, can be taken if reinforcement learning alternatives in the future are more feasible to apply for the validation component of the framework.

#### Implementation

The models that have been validated are assigned as the master instance for orchestration platforms that can manage the deployment of the models and the scalability of the implementation. Multiple models can be part of the master image, and through package management, only the relevant or necessary models are served to the individual nodes (Figure 5). Institutes and stakeholders can gain access to these models through leveled access. Version control can ensure robust and technical governance steps with audit trail of the processes and monitoring of the system performance. For example, the change of weights W can also be documented for future reference. When new candidate models are selected from the Derivation component, or if any model is ineligible due to validation, the master instance is updated. This update can then be propagated throughout the network as it is tethered to the orchestration platform. Similarly, should a model be disqualified from the framework due to technical issues or violating governance standards, the model is removed from the master instance, thereby automatically depreciating the instances at the nodes. To prioritize the deployment and maximize the utilization of resources across the nodes in the network, a reinforcement learning approach could also be integrated within this framework.

### Toward human-centered AI design

This framework is compatible with human-centered design-thinking methods^44^^,^^45^ focusing on developing solutions informed by the requirements of the stakeholders involved in developing and using the product. Furthermore, it complements systems thinking approaches to leverage points^46^^,^^47^ in the AI development pipeline that can have a meaningful impact when interacting with the healthcare network. This combination of methods, core to the architecture of this framework, would, in the long-term, lead toward a human-centered AI design practice. It can be tailored to individual implementation needs and yet create a harmonized approach toward successfully using AI-enhanced solutions at scale in the real-world context and complexity. The collaborative and multidisciplinary nature of these methods has seen recent popularity, leading to design innovation in research and practice.^33^^,^^48^

The logistics of developing and maintaining large-scale solutions include collaboration between the stakeholders. We see complications such as gaps between research and practice, lack of awareness of the levels of product-service architectures necessary in solving the design problem, or even obscurity in the levels of a system – where the development is taking place and how it interacts in a larger network.^33^ Therefore, design teams and developers involved in designing complex systems can potentially suffer from usability,^49^ and compatibility issues,^33^ fragmentation,^12^ and duplication of efforts^21^ – leading to wider issues such as increased costs, loss of trust in the system, and further complications that contribute to accountability, transparency, liability, and ethical violations.

Design teams can utilize the proposed framework when developing models and coordinating large-scale deployment, maintenance, and future updates to the system. The development of this framework started with looking at the Dimensions of Engagement (DoE) (Table 1) based on a design innovation approach by Edelman^44^ who describes that such approaches facilitate the use of performative patterns. These are micro-interactions in the design process that break down abstract cognitive tasks into smaller steps. In the context of AI models, this can be compared to the complex interaction of the many components of an algorithmic development pipeline that can now be dynamic and modular. The proposed framework further builds on this work to integrate design thinking methods that would facilitate the developers when collaborating with patients, physicians, and other stakeholders, to inherently query human-centered aspects of the development. This is particularly important when building trust with beneficiaries and utilizing resources of healthcare systems. The key to successful design outcomes involves asking analytical questions that inquire about the specifications, comparisons, and verification; asking generative questions to generate a field of possibilities.^33^^,^^50^ Although these methods could be used independently for developers and digital health designers to build systems from scratch, using the framework provides an easier architecture to follow, which is informed on the multisectoral requirements as discussed previously. Therefore, making the human-centered AI design approach more accessible and applicable for AI enhanced digital health solutions.

**Table 1:** The dimensions of engagement for xAI

Object	Context
Touch Points	Usability
• Software libraries for ML/DL (e.g., TensorFlow) • Integrated development environments (IDEs)	• Integrated ad hoc interpretability tools • Ease of access • Workflow developed for interpretability
Depth	Scenario
• Privacy preserving methods • Algorithms for ad hoc explainability	• Clinical prediction models • New evidence-base for interpretability • Reliability and robustness of models
Core	Network
• Increase interpretability of predictive models for healthcare professionals in evaluating and predicting clinical outcomes	• Interaction between different stakeholders in the healthcare sector

### Foundational steps for implementation

The burden of interpretability in healthcare still lies on the healthcare professionals, and the relevant healthcare authorities. The system that we have now, is safeguarded with regulatory oversight and governance that is constantly adapting to how innovative technologies are implemented.

Implementation of AI in healthcare is inevitable as more medical information and processes become digitized – attracting payers, providers of care, and healthcare companies.^51^ The scope in which AI can be implemented is also increasing. Not only are these emerging technologies relevant for diagnosis and treatment applications; but also, can contribute to patient engagement and adherence, and administrative applications. Although implementation issues can be challenging for healthcare systems and entities, algorithmic systems based on different ML approaches can take the field beyond simple rule-based electronic health records (EHRs).^51^ However, there are foundational steps that need to be taken to overcome wide adoption and implementation barriers. These steps include, but are not limited to, solutions toward regulatory approval and governance, better integration with existing systems, standardization with interoperability, training of clinicians and medical workforce, funding and monetization of the technology, and long-term sustainability. We could risk stagnating in a culture of prescriptive and reactive use of technology where we have problems that we want to solve with AI instead of having proactive and well-designed systems that incorporate emerging technologies to empower our systems, the users, and the beneficiaries.

As the use of AI becomes more integrated into a post-digital-transformation era, according to Maclure & Russell,^16^ resource allocation and human-centeredness – in our case, patient-centeredness – can be highly impacted if we overestimate the functionality of AI systems. The explainability of AI models is crucial to successfully implementing AI in healthcare settings. The *Health xAI Framework* is proposed as an approach to help with the digital transformation and reduce failures in implementing AI-enhanced solutions for healthcare. Using reinforcement learning techniques, where the bandit learning algorithms learn to select model combinations over time, with rewards reflecting the clinically weighted metrics. Additionally, a human-centered AI design interface utilizing personas^52^ could be developed to facilitate domain expert interactions and model selection. The framework aims to guide developers, researchers, digital health designers, and policymakers to solve health and care challenges when implementing AI at scale.

### Limitations of the framework

The proposed framework is limited in the sense that it provides a systematic approach for technical implementation but cannot be used to solve the aforementioned foundational steps. Unless we see these challenges and barriers with a holistic approach and appreciate their interconnected components, we risk fragmented and duplicated efforts that are too narrow and might miss the potential of powerful tools that can be harnessed. There are other potential limitations or challenges in implementing this framework. Implementing complex frameworks will require considerable design focus and allocation of significant resources and expertise. The integration of reinforcement learning and xAI methods requires specialized knowledge. The collaborative nature of the framework necessitates effective communication and coordination among diverse stakeholders. Additionally, there is the ongoing need to adapt to evolving regulatory landscapes and technological advancements. These potential barriers may pose challenges for certain healthcare systems, but the advantages in terms of clinical outcomes and the implementation of xAI could potentially outweigh these obstacles in the longer term. This framework provides a roadmap toward a future where AI augments human expertise in healthcare. The framework offers a structured approach to address the complexities of AI deployment in healthcare, promoting both clinical utility and human-centered design. To assess the effectiveness of the proposed framework and to understand its impact, future evaluation and comparative studies can generate knowledge about best practices from its implementation.

## Acknowledgments

This research was funded by the 10.13039/501100000272National Institute for Health and Care Research (10.13039/501100000272NIHR) Applied Research Collaboration Oxford and Thames Valley, at 10.13039/501100023234Oxford Health NHS Foundation Trust.

The views expressed are those of the author(s) and not necessarily those of the NHS, the NIHR or the Department of Health and Social Care.

## Author contributions

ANSI/NISO Z39.104–2022, CRediT, Contributor Roles Taxonomy.

H.S.A.: Conceptualization, funding acquisition, investigation, methodology, validation, visualization, writing – original draft, writing – review and editing; A.S.: conceptualization, methodology, validation, writing – review and editing; L.C.: supervision, writing – review and editing; C.B.: supervision, writing – review and editing; R.P.-S.: supervision, writing – review and editing.

## Declaration of interests

The authors declare no competing interests.
